# Sex and Gender Equity in Research: rationale for the SAGER guidelines and recommended use

**DOI:** 10.1186/s41073-016-0007-6

**Published:** 2016-05-03

**Authors:** Shirin Heidari, Thomas F. Babor, Paola De Castro, Sera Tort, Mirjam Curno

**Affiliations:** 1 EASE Gender Policy Committee/Reproductive Health Matters, London, UK; 2 Department of Community Medicine, University of Connecticut School of Medicine, Farmington, CT 06030-6325 USA; 3 Istituto Superiore di Sanità, Rome, Italy; 4 Cochrane Editorial Unit, London, UK; 5 Journal of the International AIDS Society, Geneva, Switzerland

**Keywords:** Sex, Gender, Guidelines, SAGER, Scientific research, Scientific publishing, Gender bias, Equity

## Abstract

**Background:**

Sex and gender differences are often overlooked in research design, study implementation and scientific reporting, as well as in general science communication. This oversight limits the generalizability of research findings and their applicability to clinical practice, in particular for women but also for men. This article describes the rationale for an international set of guidelines to encourage a more systematic approach to the reporting of sex and gender in research across disciplines.

**Methods:**

A panel of 13 experts representing nine countries developed the guidelines through a series of teleconferences, conference presentations and a 2-day workshop. An internet survey of 716 journal editors, scientists and other members of the international publishing community was conducted as well as a literature search on sex and gender policies in scientific publishing.

**Results:**

The Sex and Gender Equity in Research (SAGER) guidelines are a comprehensive procedure for reporting of sex and gender information in study design, data analyses, results and interpretation of findings.

**Conclusions:**

The SAGER guidelines are designed primarily to guide authors in preparing their manuscripts, but they are also useful for editors, as gatekeepers of science, to integrate assessment of sex and gender into all manuscripts as an integral part of the editorial process.

## Background

Sex and gender are important determinants of health and well-being. Sex refers to a set of biological attributes in humans and animals that are associated with physical and physiological features including chromosomes, gene expression, hormone function and reproductive/sexual anatomy [1]. Sex is usually categorized as female or male, although there is variation in the biological attributes that constitute sex and how those attributes are expressed.

Gender refers to the socially constructed roles, behaviours and identities of female, male and gender-diverse people [1]. It influences how people perceive themselves and each other, how they behave and interact and the distribution of power and resources in society. Gender is usually incorrectly conceptualized as a binary (female/male) factor. In reality, there is a spectrum of gender identities and expressions defining how individuals identify themselves and express their gender. A glossary of terms is provided in Appendix 1 to define the meaning of sex, gender and related terms.

Sex and gender interactions influence health and well-being in a variety of ways. They both impact environmental and occupational risks, risk-taking behaviours, access to health care, health-seeking behaviour, health care utilization, and perceived experience with health care, and thus disease prevalence and treatment outcome. In addition, it is well-known that pharmacokinetics and pharmacodynamics of pharmaceutical agents differ between sexes, resulting in differential adverse event profiles and further impacting treatment outcomes. Thus, sex and gender are critical determinants of health [2].

### Sex and gender bias in the conduct of research

Despite recognition of the importance of sex and gender in most areas of research, important knowledge gaps persist owing to the general orientation of scientific attention to one sex or gender category and because of a misconception that disaggregation of sex does not apply to other living organisms that can be classified by sex [3–6].

The gap in the representation of women in studies on human subjects has been well-documented [1]. A review of cardiovascular treatment trials included in Cochrane Reviews reveals that only 27 % of the total trial participants in the 258 clinical trials were women [7]. More importantly, among trials recruiting both men and women, only one third reported a gender-based analysis [8]. More than 79 % of animal studies published in *Pain* over a 10-year period included male subjects only, and only 4 % studied sex differences [9].

The underrepresentation of women in research can result in adverse consequences. Among the ten prescription pharmaceuticals withdrawn from the US market between 1997 and 2001, eight caused greater harm to women than men [10]. More recently, the US Food and Drug Administration (FDA) issued a safety communication, recommending half a dose of zolpidem for women, due to greater susceptibility to the risks of the drug [11]. Sex- and gender-based analysis, in all of these cases, would have provided sufficient information to guide dosing and applicability of drugs in men and women prior to approval.

Failure to conduct sex and gender-based analysis occurs in a range of disciplines. In the field of engineering, lack of consideration of differences in the physiology and anatomy of males and females in developing car seats has resulted in higher risk for whiplash injuries among female car occupants compared with men [12, 13].

Although the term “gender gap” has most often been applied to women, the benefit that sex- and gender-based analysis has for our understanding of men’s health should also be noted. Despite the increasing representation of male and female subjects in research and reporting of sex-specific and gender-specific data, these examples indicate that existing policies have not been enforced [3]. Lack of interest in sex and gender differences may not only be harmful but also present missed opportunities for innovation. Understanding the underlying differences and similarities, exploring applicability, uptake and impact of technological innovations and getting deeper insight into cognitive variability will undoubtedly lead to more innovative approaches and better solutions to meet the needs of society.

### The role of journal editors and editorial policies

Editors play an important role as gatekeepers of science, including the articulation of an ethical framework that influences the conduct of research. With an ever-increasing volume of information being published, concerns over the quality of publications have lead journal editors, publishers and professional associations to implement detailed guidelines. Ethical review procedures are now universally applied in human and animal research, in part because of journal requirements. The impact of journal policies on compliance to mandates has been clearly demonstrated in such diverse areas as clinical trial registration [14] and the reporting of systematic reviews after introduction of PRISMA guidelines [15]. Another illustration is the gradual adoption of the Consolidated Standards of Reporting Trials (CONSORT) statement, which has led to improved reporting of randomized controlled trials [16, 17]. Following CONSORT and PRISMA, many other reporting guidelines have been developed, including the ARRIVE guidelines for animal research [18].

Although policy implementation and enforcement continue to be a critical challenge, journals could play an important role in advancing the quality and transparency of reported data by promoting sex- and gender-specific analysis of research data as a matter of routine. In a 2011 workshop on “Sex-specific reporting of scientific research,” convened by the US Institute of Medicine, a number of key issues were identified that journals and journal editors should address in order to improve gender-sensitive reporting of research [3], including the appropriateness of sex-specific data analyses and the absence of journal policies recommending sex and gender considerations in research design and reporting.

On the basis of the available evidence, a committee of the US Institute of Medicine in 2010 recommended that the International Committee of Medical Journal Editors (ICMJE) and other editors adopt a guideline that all papers reporting the results of clinical trials analyse data separately for men and women. The ICMJE has since published more robust guidance on sex and gender reporting, recommending that researchers include representative populations in all study types, provide descriptive data for sex and other relevant demographic variables and stratify reporting by sex [19].

Adequate inclusion of sufficient numbers of men and women (and other sub-populations) in research, along with appropriate analysis and transparent and complete reporting of research data, require a concerted effort among funders, researchers, reviewers and editors [20]. Although editors typically enter the research process late, after the research has already been concluded and the data analysed, they can still play an important role in ensuring effective, transparent and complete sex and gender reporting.

In recent years, several reviewers of sex and gender issues in scientific research have made recommendations regarding the best ways to address the problems that have been identified. Doull et al. [21, 22] proposed that the methodology of systematic reviews and of sex- and gender-based analyses be refined and synchronized to enhance the collection, synthesis and analysis of evidence for decision-making, and they developed an appraisal tool for systematic reviews and adapted it to evaluate primary studies and protocols for new research [22]. Nowatski and Grant [23] provided a rationale for gender-based analysis, which is designed to identify the sources and consequences of inequalities between women and men and to develop strategies to address them. The Clinical Orthopedics and Research journal published an editorial on gender and sex in scientific reporting in 2014, including a set of recommendations [5].

Editorial associations, publishing houses, funding agencies and public interest organizations have also taken an interest in sex and gender issues. The Canadian Institutes of Health Research implemented a requirement in 2010 that all grant applicants respond to mandatory questions about whether their research designs include gender and sex [24]. Advances made in the inclusion of women as research participants in the USA can be attributed in large part to the actions taken at the NIH in 1993 that stipulated women and minorities should be included in phase 3 clinical trials so that valid analyses of differences in intervention effects could be performed [25]. More recently, the NIH announced plans to require grant applicants to describe how they will balance of male and female cells and animals in preclinical studies, unless sex-specific inclusion is unwarranted [6].

Despite a greater recognition of the importance of sex and gender considerations in research and scientific publishing, progress has been slow in some areas of science and further work is needed to build on preceding efforts by journals, journal editors and learned societies. As noted by Nieuwenhoven [26], vigorous approaches are needed to stimulate scientists to integrate sex and gender aspects into their research. For example, there is no overarching set of recommendations that provides guidelines for better reporting of sex and gender in scientific publications across disciplines. To address this need, the present article describes the development of a set of international guidelines to encourage a more systematic approach to the reporting of sex and gender in research across disciplines.

## Methods

The European Association of Science Editors (EASE) established a Gender Policy Committee in 2012 and tasked it to develop a set of guidelines for reporting of Sex and Gender Equity in Research (SAGER). A panel of 13 experts (eight females, five males) representing nine countries were selected by the Chairperson of the GPC (Dr. Heidari). Eight members were senior editors for a variety of biomedical journals, and the remaining individuals had expertise on gender research and scientific publishing.

An internet survey of 716 journal editors, scientists and other members of the international publishing community was first conducted to gather information about existing sex and gender policies and opinions about the need for such policies. The survey focused on four policy areas: (1) instructions for authors that require or encourage disaggregation of data by sex or gender when feasible; (2) gender policies concerning the composition of editorial staff and boards; (3) policies that strive for gender balance among peer reviewers and (4) guidelines that ask reviewers to assess manuscripts for inclusion of sex-disaggregated data and gender analysis. The survey targeted four groups: EASE members; members of the International Society of Addiction Journal Editors (ISAJE); a random sample of 100 journals selected from the 8607 names in the Thomson Reuters SCI Expanded database of journals and an open sample in which any concerned individual could complete the survey. In total, 716 respondents took part in the survey, representing 338 unique journals and 114 unique publishing houses.

In addition to the survey, several other methods were used to identify policy options and expert recommendations. First, keyword searches were conducted (e.g. “sex” + “instructions for authors”) to identify journals that had specific policies on sex and gender. In addition, we scanned the websites of surveyed journals that explicitly expressed concerns about sex and gender knowledge gaps in science and the sex and gender reporting policies of peer-reviewed journals already known to the Gender Policy Committee.

Over a 3-year period, the Committee worked through a series of teleconferences, conference presentations and a 2-day workshop to develop its recommendations. Once the draft guidelines were developed, dissenting views were considered at editors’ meetings in Blankenberge, Belgium, and Split, Croatia. In addition, the draft guidelines were circulated to 36 experts in sex and gender research; any comments received were incorporated into the document where relevant.

## Results

### Survey findings

The average proportion of respondents in each of the four samples who reported having sex and/or gender policies at their journals was 7 %. Respondents from countries where men and women are more equal (lower GII) were more likely to report that these policies are in place.

In the random sample of 100 journals and the EASE and ISAJE groups, a majority (75 %) were unsure or unwilling to introduce sex and gender considerations as requirements in Instructions for Authors. Female respondents were more likely to support sex and gender reporting policies than male respondents. While caution must be exercised in relation to the conclusions drawn, the survey results point to the paucity of sex- and gender-related policies concerning instructions for authors, guidelines for peer-reviewers and gender balance of both editorial boards and peer-reviewers.

### Literature review

Our review identified policies developed and used by 62 journals, as well as 25 other sources of published materials in the form of journal articles, editorials, expert committee reports and conference proceedings.

The majority of sex and gender policies and guidelines fell into the Instructions for Authors category, covering a variety of scientific areas (e.g. “animal science,” “health – psychiatry”) and types of research (e.g. animal, human, cell or a combination of the three). In most cases, the instructions merely advise authors to report results for males and females separately, if appropriate.

Several journals [20, 27, 5] have used their editorial pages to announce the adoption of new policies or to promote the need for greater awareness of sex and gender issues. For example, the editors of Clinical Orthopaedic and Related Research published an editorial [5] recommending that researchers seeking publication in the journal use the following guidelines: (1) design studies that are sufficiently powered to answer research questions both for males and females if the health condition being studied occurs in all sexes and genders; (2) provide sex- and/or gender-specific data where relevant in all clinical, basic science and epidemiological studies; (3) analyse the influence (or association) of sex or gender on the results of the study, or indicate in the “Methods” section why such analyses were not performed, and consider this topic as a limitation to cover in the “Discussion” section and (4) if sex or gender analyses were performed post hoc, indicate that these analyses should be interpreted cautiously.

In a 2011 workshop “Sex-specific reporting of scientific research,” a broad cross section of stakeholders convened by the US Institute of Medicine identified key issues that journals and journal editors should address, such as requiring authors to report on the sex of study subjects, not only in studies with human participants but also in animal research and in studies with cells, tissues and other material from humans or animals.

Doull et al. [21] proposed that the methodologies of systematic reviews and of sex- and gender-based analysis be refined and synchronized to enhance the collection, synthesis and analysis of evidence for decision-making. Nowatski and Grant [23] provided a rationale for gender-based analysis (GBA), which is designed to identify the sources and consequences of inequalities between women and men and to develop strategies to address them. GBA focuses on gender differences in health and health care and appropriate policies.

### SAGER guidelines

The policies, procedures and recommendations reviewed above were used as a basis for the SAGER guidelines, which are designed to promote systematic reporting of sex and gender in research. The guidelines provide researchers and authors with a tool to standardize sex and gender reporting in scientific publications, whenever appropriate. They are also aimed at editors to use as a practical instrument to evaluate a research manuscript and as a vehicle to raise awareness among authors and reviewers. Although reporting guidelines typically focus on how to report what was actually done in a study, we recognize that not all of the items included in the SAGER guidelines are feasible or applicable to a particular study. For this reason, SAGER encourages authors, editors and referees to consider if sex and gender are relevant to the topic of the study, and accordingly to follow the guidelines, whenever applicable. As a general principle, the SAGER guidelines recommend careful use of the words sex and gender in order to avoid confusing both terms. The use of common definitions will improve the ability to conduct meta-analyses of published and archived data. The term sex should be used as a classification of male or female based on biological distinction to the extent that this is possible to confirm. Authors should underline in the methods section whether sex of participants was defined based on self-report, or assigned following external or internal examination of body characteristics, or through genetic testing or other means. In studies of animals, the term sex should be used. In cell biological, molecular biological or biochemical experiments, the origin and sex chromosome constitutions of cells or tissue cultures should be stated. If unknown, the reasons should be stated. In other disciplines, such as the testing of devices or technology, authors should explain whether it will be applied or used by all genders and if it has been tested with a user’s gender in mind.

It is acknowledged that many studies will not have been “designed” to analyse sex and/or gender differences, but the panel felt these analyses are necessary to advance knowledge about sex and gender, especially in medical research.

Table 1 presents the SAGER guidelines. They apply to all research with humans, animals or any material originating from humans and animals (e.g. organs, cells, tissues), as well as other disciplines whose results will be applied to humans such as mechanics and engineering.

**Table 1:** Sex and Gender Equity in Research (SAGER) guidelines

General principles	
• Authors should use the terms *sex* and *gender* carefully in order to avoid confusing both terms.	
• Where the subjects of research comprise organisms capable of differentiation by sex, the research should be designed and conducted in a way that can reveal sex-related differences in the results, even if these were not initially expected.	
• Where subjects can also be differentiated by gender (shaped by social and cultural circumstances), the research should be conducted similarly at this additional level of distinction.	
Recommendations per section of the article	
Title and abstract	If only one sex is included in the study, or if the results of the study are to be applied to only one sex or gender, the title and the abstract should specify the sex of animals or any cells, tissues and other material derived from these and the sex and gender of human participants.
Introduction	Authors should report, where relevant, whether sex and/or gender differences may be expected.
Methods	Authors should report how sex and gender were taken into account in the design of the study, whether they ensured adequate representation of males and females, and justify the reasons for any exclusion of males or females.
Results	Where appropriate, data should be routinely presented disaggregated by sex and gender. Sex- and gender-based analyses should be reported regardless of positive or negative outcome. In clinical trials, data on withdrawals and dropouts should also be reported disaggregated by sex.
Discussion	The potential implications of sex and gender on the study results and analyses should be discussed. If a sex and gender analysis was not conducted, the rationale should be given. Authors should further discuss the implications of the lack of such analysis on the interpretation of the results.

#### Title and abstract

If only one sex or gender is included in the study, the title and the abstract should specify the sex of animals or any cells, tissues and other material derived from these and the sex and gender of human participants. In applied sciences (technology, engineering, etc.), authors should indicate if the study model was based on one sex or the application was considered for the use of one specific sex. For studies (of a non-sex-specific issue) in which only one sex has been used, the article’s title should specify this fact by including “in males” or “in females” in the title and abstract. If cultures of primary cells, tissue, etc., were obtained from one sex, the sex should be indicated in the title [3].

#### Introduction

Authors should report, where relevant, previous studies that show presence or lack of sex or gender differences or similarities. If such studies are lacking, the authors should explain whether sex and/or gender may be an important variant and if differences may be expected.

#### Methods

Authors should report how sex and gender were taken into account in the design of the study, ensure adequate representation of males and females and justify reasons for the exclusion of males or females. Methodological choices about sex and gender in relation to study population and analytical approach should be reported and justified in the same way as other methodological choices.

In vivo and in vitro studies using primary cultures of cells, or cell lines from humans or animals, or ex vivo studies with tissues from humans or animals must state the sex of the subjects or source donors, except for immortalized cell lines, which are highly transformed [3]. In other cases, e.g. embryonic or early postnatal cultures, cell lines immortalized from a mixed culture or previously completed experiments for which sex was not documented, it is recommended that researchers determine the sex of cells or cell lines by chromosomal analysis and that the designations “mixed” or “unknown” should be used only when the sex cannot be determined through any methods.

#### Results

Data should be reported disaggregated by sex, and an analysis of sex and gender differences and similarities should be described, where appropriate. Anatomical and physiological differences between men and women (height, weight, body mass, cell counts, hormonal cycles, etc.) as well as social and cultural variables (socio-economic status, education, etc.) should be taken into consideration in the presentation of data and/or analysis of the results. We recommend the use of the gendered innovations’ checklist for animals, tissues, cells and cultures [28]. If sex- and gender-based analyses have been performed, results should be reported regardless of the positive or negative outcome. In human studies, data on enrolment, participation, dropout, discontinuation and loss-to-follow up should be reported disaggregated by sex and gender (where appropriate), and the influence of sex and gender factors should be assessed a priori on the basis of their hypothesized role in the causation, course, treatment effectiveness, impact and outcome of health problems. Authors should refrain from conducting a post hoc gender-based analysis if the study design is insufficient to enable meaningful conclusions. In all cases, raw data should be published disaggregated by sex and gender for future pooling and meta-analysis.

In epidemiological studies, the impact of other exposures, such as socioeconomic variables, on health problems should be examined for all genders and should be analysed critically from a gender perspective.

We recognize that reporting guidelines focus on how to report what was actually done. However, not all of the items in the SAGER guidelines need to be done, as indicated by the words, “if appropriate.” The SAGER guidelines are intended to promote sex and gender equity in research; therefore, it encourages authors, editors and referees to consider if sex and gender are relevant to the topic of the study, and accordingly to follow the guidelines, whenever applicable.

#### Discussion

The implications of sex and gender for the interpretation of study results should be elaborated, including the extent to which the findings can be generalized to all sexes and genders in a population. If no sex and gender-based analyses have been performed, authors should indicate the reasons for lack of such analyses when discussing the limitations of the study and discuss whether such analyses could have affected the results.

When interpreting research findings, past research should be examined for both methodological rigour and sex bias in procedure and interpretation. Authors should avoid confusing sex with gender and reducing complex or interactionist explanations to overly simple ones. Authors should consider all possible explanations for sex- and gender-related phenomena including social, cultural, biological and situational factors, recognizing that many sex-related behaviours might result from either cultural factors or biological factors. Covariation between biology and behaviour does not constitute evidence for physiological causation.

Appendix 2 provides a set of questions intended to raise awareness among authors. For many disciplines engaged in original scientific research, this list could serve as a basis for the preparation of a manuscript for submission.

## Conclusions

The SAGER guidelines were developed over a 3-year period by a multidisciplinary group of academics, scientists and journal editors by means of literature reviews, expert feedback and public consultations at conferences. Authors, journal editors, publishers, reviewers and other members of the scientific community all have a role to play in addressing the neglect of the sex and gender dimension in scientific publishing.

The SAGER guidelines provide researchers and authors with a tool to standardize sex and gender reporting in scientific publications. They were designed to improve sex and gender reporting of scientific research, serve as a guide for authors and peer-reviewers, be flexible enough to accommodate a wide range of research areas and disciplines and improve the communication of research findings. Nevertheless, the guidelines do not make explicit recommendations regarding gender-diverse populations. We recognize that most studies will not be powered to detect differences in effects for gender-diverse populations such as transgender, especially in countries where such diversity is unknown. Yet authors need to consider the relevance of their research for gender-diverse populations.

**Fig. 1:**
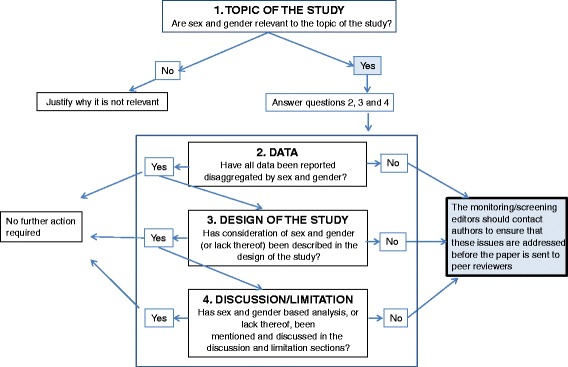
SAGER flowchart guiding editors’ initial screening of submitted manuscripts

Editors should make it clear that integration of sex and gender issues makes for more rigorous and ethical science. To the extent that mandates are difficult to implement, we recommend that journal editors endorse the SAGER guidelines and adapt them to the needs of their journals and their fields of science by including examples of good practice for each of the reporting items. At a minimum, journals publishing original research should request in their instructions to authors that all papers present data disaggregated by sex and gender and, where applicable, explain sex and gender differences or similarities adequately. Figure 1 provides a list of questions that could be used to guide the initial screening of submitted manuscripts. Editors should introduce specific questions in the checklist used to screen initial submissions, as an effort to systematize gender-conscious assessment of manuscripts among editorial staff. The following is an example of questions that can be introduced in peer-reviewers’ assessment forms:

1. Are sex and gender relevant to the research in question?

2. Have authors adequately addressed sex and gender dimensions or justified absence of such analysis?

To be effective, the guidelines need to be endorsed by a broad cross section of the scientific community, including journal editors, publishers, editors’ societies, professional organizations, scientific advocacy groups, science journalists and other science communicators.

Editors should distribute the SAGER guidelines to their reviewers and encourage them to use them in the evaluation of manuscripts. They should ensure the manuscript assessment forms completed by peer-reviewers include specific questions regarding the importance and relevance of sex and gender.

Training the editorial staff on the importance of sex and gender-sensitive reporting should be conducted as part of regular training on ethical conduct and editorial practices.

## Appendix 1

### Glossary of terms

*Gender*. Gender refers to the socially constructed roles, behaviours and identities of female, male and gender-diverse people [1]. It influences how people perceive themselves and each other, how they behave and interact and the distribution of power and resources in society. Gender is usually incorrectly conceptualized as a binary factor (female/male). In reality, there is a spectrum of gender identities and expressions defining how individuals identify themselves and express their gender.

*Gender identity*. A person’s concept of self as being male and masculine or female and feminine, or ambivalent, based in part on physical characteristics, parental responses and psychological and social pressures. It is the internal experience of gender role. (Mesh term, introduced in 1991, revised in 1975).

*Gender-based analysis*. An analytical tool that systematically integrates a gender perspective into the development of policies, programmes and legislation, as well as planning and decision-making processes. It helps to identify and clarify the differences between women and men and boys and girls and demonstrates how these differences affect health status, access to, and interaction with, the health care system.

(http://www.hc-sc.gc.ca/hl-vs/pubs/women-femmes/gender-sexes-eng.php)

*Gender-sensitive analysis*. Analysis of statistics that goes beyond simply disaggregating data according to sex (e.g. a mere “sex-counting” is not sufficient). Gender-sensitive analysis should question the underlying gender relations which are reflected in the data.

(http://www.oecd.org/dac/gender-development/44896238.pdf)

*Gender perspective*. The gender perspective looks at the impact of gender on people’s opportunities, social roles and interactions. Successful implementation of the policy, programme and project goals of international and national organizations is directly affected by the impact of gender and, in turn, influences the process of social development. Gender is an integral component of every aspect of the economic, social, daily and private lives of individuals and societies and of the different roles ascribed by society to men and women.

(http://www.fao.org/docrep/003/x2919e/x2919e04.htm)

*Sex.* Sex refers to a set of biological attributes in humans and animals that are associated with physical and physiological features including chromosomes, gene expression, hormone function and reproductive/sexual anatomy [1]. Sex is usually categorized as female or male, although there is variation in the biological attributes that constitute sex and how those attributes are expressed.

*Sex- and Gender-Based Analysis*. An analytical approach that integrates a sex and gender perspective into the development of health research, policies and programmes, as well as health planning and decision-making processes. It helps to identify and clarify the differences between women and men and boys and girls and demonstrates how these differences affect health status, access to, and interaction with, the health care system.

(http://www.hc-sc.gc.ca/hl-vs/gender-genre/analys/index-eng.php)

*Sex-disaggregated data*. Data that are collected and presented separately on men and women. Gender Mainstreaming Implementation Framework—UNESCO, 2003.

*Sexism*. Prejudice or discrimination based on gender or behaviour or attitudes that foster stereotyped social roles based on gender. (MESH term, introduced in 2013).

*Transgender Persons, Transexual persons, Transgenders*. Persons having a sense of persistent identification with, and expression of, gender-coded behaviours not typically associated with one’s anatomical sex at birth and with or without a desire to undergo sex reassignment procedures (MeSH term 2016 (2013).

## Appendix 2

**Table 2:** Authors’ checklist for gender-sensitive reporting

Research approaches ✓	
	✓ Are the concepts of gender and/or sex used in your research project?
	✓ If yes, have you explicitly defined the concepts of gender and/or sex? Is it clear what aspects of gender and/or sex are being examined in your study?
	✓ If no, do you consider this to be a significant limitation? Given existing knowledge in the relevant literature, are there plausible gender and/or sex factors that should have been considered? If you consider sex and/or gender to be highly relevant to your proposed research, the research design should reflect this
Research questions and hypotheses	
	✓ Does your research question(s) or hypothesis/es make reference to gender and/or sex, or relevant groups or phenomena? (e.g., differences between males and females, differences among women, seeking to understand a gendered phenomenon such as masculinity)
Literature review	
	✓ Does your literature review cite prior studies that support the existence (or lack) of significant differences between women and men, boys and girls, or males and females?
	✓ Does your literature review point to the extent to which past research has taken gender or sex into account?
Research methods	
	✓ Is your sample appropriate to capture gender and/or sex-based factors?
	✓ Is it possible to collect data that are disaggregated by sex and/or gender?
	✓ Are the inclusion and exclusion criteria well justified with respect to sex and/or gender? (Note: this pertains to human and animal subjects and biological systems that are not whole organisms)
	✓ Is the data collection method proposed in your study appropriate for investigation of sex and/or gender?
	✓ Is your analytic approach appropriate and rigorous enough to capture gender and/or sex-based factors?
Ethics	
	✓ Does your study design account for the relevant ethical issues that might have particular significance with respect to gender and/or sex? (e.g., inclusion of pregnant women in clinical trials)
*Source: Adapted from Canadian Institutes of Health Research.*	
